# Karyotypic description of the stingless bee *Meliponaquinquefasciata* Lepeletier, 1836 (Hymenoptera, Meliponini) with emphasis on the presence of B chromosomes

**DOI:** 10.3897/CompCytogen.v12i4.29165

**Published:** 2018-11-09

**Authors:** Alexandra Avelar Silva, Marla Piumbini Rocha, Silvia das Graças ompolo, Lucio Antonio de Oliveira Campos, Mara Garcia Tavares

**Affiliations:** 1 Departamento de Biologia Geral, Universidade Federal de Viçosa, Viçosa, MG, 36570-000, Brazil Universidade Federal de Viçosa Viçosa Brazil; 2 Departamento de Morfologia, Universidade Federal de Pelotas, Pelotas, RS, 96030-000, Brazil Universidade Federal de Pelotas Pelotas Brazil

**Keywords:** Cytogenetics, heterochromatin, karyotype, fluorochromes, FISH

## Abstract

Stingless bees are distributed widely in the tropics, where they are major pollinators of several plant species. In this study, the karyotype of *Meliponaquinquefasciata* Lepeletier, 1836 was analysed, with emphasis on the presence of B chromosomes. Post-defecating larvae were analysed using Giemsa staining, the C-banding technique, sequential staining with fluorochromes, and FISH. The chromosome number ranged from 2n = 18 to 22 (females) and from n = 9 to 13 (males) due to the presence of 0–4 B chromosomes. This result demonstrates that *M.quinquefasciata* has the same chromosomal number as other *Melipona* Illiger, 1806 species. Considering the A complement, heterochromatin was located only in the pericentromeric region of pair 1. Staining with chromomycin A_3_ (CMA_3_) and labelling with rDNA probe, indicated that this region corresponded to the nucleolus organising region. The B chromosomes of *M.quinquefasciata* could be found in individuals from different localities, they were completely heterochromatic (C-banding) and uniformly stained by 4’,6-diamidino-2-phenylindole (DAPI). Variations in the number of B chromosomes were detected between cells of the same individual, between individuals of the same colony, and between colonies from different localities.

## Introduction

Classical or molecular cytogenetic analysis can be used to determine chromosome number and morphology, the location and quantity of AT or CG rich regions, nucleolus organizing regions, rRNA clusters and repetitive sequences in the genome. This information allows species characterization, identification of cryptic species and the mechanisms involved in their speciation, analysis of population variability, and studies on karyotype evolution, phylogeny and taxonomy of different groups of species (Rocha and Pompolo 1998, Lachowska et al. 2009, Mendes-Neto et al. 2010, Panzera et al. 2012, Mandrioli et al. 2014, Golub et al. 2016).

Such analysis can also identify intra-specific or numerical variations within a population due to the presence of B or extra chromosomes (Brito et al. 1997, Tosta et al. 2004, Martins et al. 2014). These chromosomes are usually heterochromatic, smaller than the normal complement chromosomes, and show a non-Mendelian segregation pattern. They have already been described in many animal and plant species, allowing for studies on their origin, stability and maintenance (Camacho 2005, Houben et al. 2014, Anjos et al. 2016).

In the order Hymenoptera, the presence of B chromosomes have already been reported in ants, wasps and bees. In ants, these chromosomes were detected in species of several genera (Lorite et al. 2002, Mariano et al. 2001, reviewed by Loiselle et al. 1990 and Gokhman 2009). In the parasitoid wasps, until now, these chromosomes were only found in *Nasoniavitripennis* Walker, 1836 (Pteromalidae), *Trichogrammakaykai* Pinto et Stouthamer, 1997 (Trichogrammatidae), *Encarsiaasterobemisiae* Viggiani et Mazzone, 1980 (Aphelinidae) and in *Pnigalioagraules* Walker, 1830, *P.gyamiensis* Myartseva et Kurashev, 1990 and *P.mediterraneus* Ferrière & Delucchi, 1957 (Eulophidae) (Nur et al. 1988, Baldanza et al. 1999, Stouthamer et al. 2001, Gebiola et al. 2012, Gokhman et al. 2014). B chromosomes have also been identified in *Trypoxylonalbitarse* Fabricius, 1804 (Crabronidae) (Araújo et al. 2000). Finally, in bees, B chromosomes have been reported in the genera *Melipona* Illiger, 1806 (*M.rufiventris* Lepeletier, 1836 and *M.quinquefasciata* Lepeletier, 1836), *Partamona* Schwarz, 1939 (*P.cupira* Smith, 1863, *P.helleri* Friese, 1900 and *P.rustica* Pedro et Camargo, 2003) and *Tetragonisca* Moure, 1946 (*T.fiebrigi* Schwarz, 1938) (revision in Tavares et al. 2017). They are also probably present in the species *P.criptica* Pedro et Camargo, 2003, *P.seridoensis* Pedro et Camargo, 2003, *P.gregaria* Pedro et Camargo, 2003, *P.chapadicola* Pedro et Camargo, 2003 and P.aff.helleri since molecular analysis demonstrated the presence of a sequence-characterized amplified region (SCAR) marker specific to the B chromosome of *P.helleri* in these genomes (Correia et al. 2014, Tosta et al. 2014, Machado et al. 2016). However, for these species, the presence of B chromosomes needs to be confirmed through cytogenetic techniques, as does the variation found in the sawfly *Tenthedrobrevicornis* (Konow, 1886) (Sanderson 1970) and in the Braconidae, *Aphidiuservi*, Halliday, 1834 (Gokhman and Westendorff 2003).

The number of species with B chromosomes, however, increases as new species are studied cytogenetically (Camacho et al. 2000). For example, for many years it was considered that *M.quinquefasciata* had n = 18 and, consequently, 2n = 36 (Kerr 1972), a diploid number very different from that of most *Melipona* species surveyed so far (n = 9 and 2n = 18; revision in Tavares et al. 2017). However, Kerr (1972) probably examined a colony that was yielding diploid males (Tarelho 1973). Then, Pompolo (1992) reported that analysis of one colony of *M.quinquefasciata* showed 2n = 20 chromosomes. It was only when a cytogenetic analysis was carried out several years later that *M.quinquefasciata* was found to have the same chromosome number as the majority of other *Melipona* species, 2n = 18, and that the numeric variations found in the karyotype of this species (2n = 19–22 and n = 9–13) were attributed to the presence of different numbers of supernumerary chromosomes (Rocha 2002, Rocha et al. 2007). However, despite comparing the general characteristics of the karyotype of *M.quinquefasciata* with that of other *Melipona* species, Rocha et al. (2007) did not specifically described the karyotype of *M.quinquefasciata*, the banding patterns obtained, or the variation in the number of B chromosomes found.

Thus, in the present study, we combined the data obtained by Rocha (2002) for two colonies of *M.quinquefasciata* with the analysis of five other colonies in order to: 1) describe in detail the karyotype of *M.quinquefasciata*, including the chromosome number, morphology and the location of heterochromatic regions, regions rich in AT/CG and ribosomal genes, and (2) verify the existence of B chromosomes in colonies from different locations, as well as their variation within colonies.

## Materials and methods

### Biological material

Post-defecating *M.quinquefasciata* larvae obtained from a colony from Brasília, DF (15°46'47"S, 47°55'47"W) and one from Luziânia, GO (16°15'09"S, 47°57'01"W) were analysed in 2000–2002 (Rocha 2002). Later, in 2013, we analysed three more colonies from Bicas, MG (21°43'31"S, 43°03'34"W), and two from Januária, MG (15°29'17"S, 44°21'42"W; State Park of Veredas of Peruaçú, PEVP).

### Chromosome preparation and treatments

Chromosome preparations (Imai et al. 1988) were obtained using cerebral ganglion cells of larvae in the final stage of defecation. The number of individuals and number of metaphases per individual analysed varied from colony to colony (Suppl. material 1: Table S1).

To determine the number and morphology of the chromosomes, conventional staining was performed using Giemsa diluted in Sorensen buffer at a ratio of 1:30, for 20 minutes. The C-banding technique was used for heterochromatin detection (Rocha and Pompolo 1998). Metaphases were analysed on an Olympus BX60 microscope and the karyotypes were assembled using Image-Pro Plus (Version 6.3, Media Cybernetics 2009). The chromosomes were classified according to Levan et al. (1964), and the karyotypes were arranged by pairing chromosomes in decreasing order of size.

Sequential staining with fluorochromes 4’,6-diamidino-2-phenylindole (DAPI) and chromomycin A_3_ (CMA_3_) was performed according to Schweizer (1980), using DAPI first for 30 min, followed by CMA_3_ for 1 h. The use of distamycin was omitted. The fluorescent *in situ* hybridisation (FISH) technique (Viegas-Pequignot 1992) was performed using the 45S rDNA probe pDm 238 (Roiha et al. 1981). The best images were captured by a CCD camera coupled to an Olympus BX-60 epifluorescence microscope, using excitation filters WB (λ = 330–385 nm) and WU (λ = 450–480 nm), under immersion and at 100× magnification.

## Results and discussion

**Figure 1.**
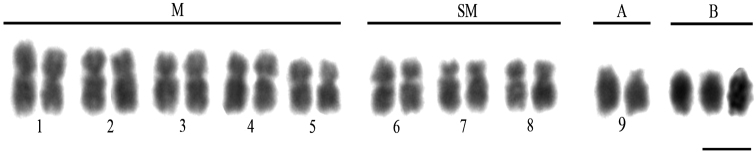
Representative karyotype of *Meliponaquinquefasciata* female, with three B chromosomes, stained with Giemsa. M, SM, A and B: metacentric, submetacentric, acrocentric and B chromosomes, respectively. Scale bar: 5μm.

**Figure 2.**
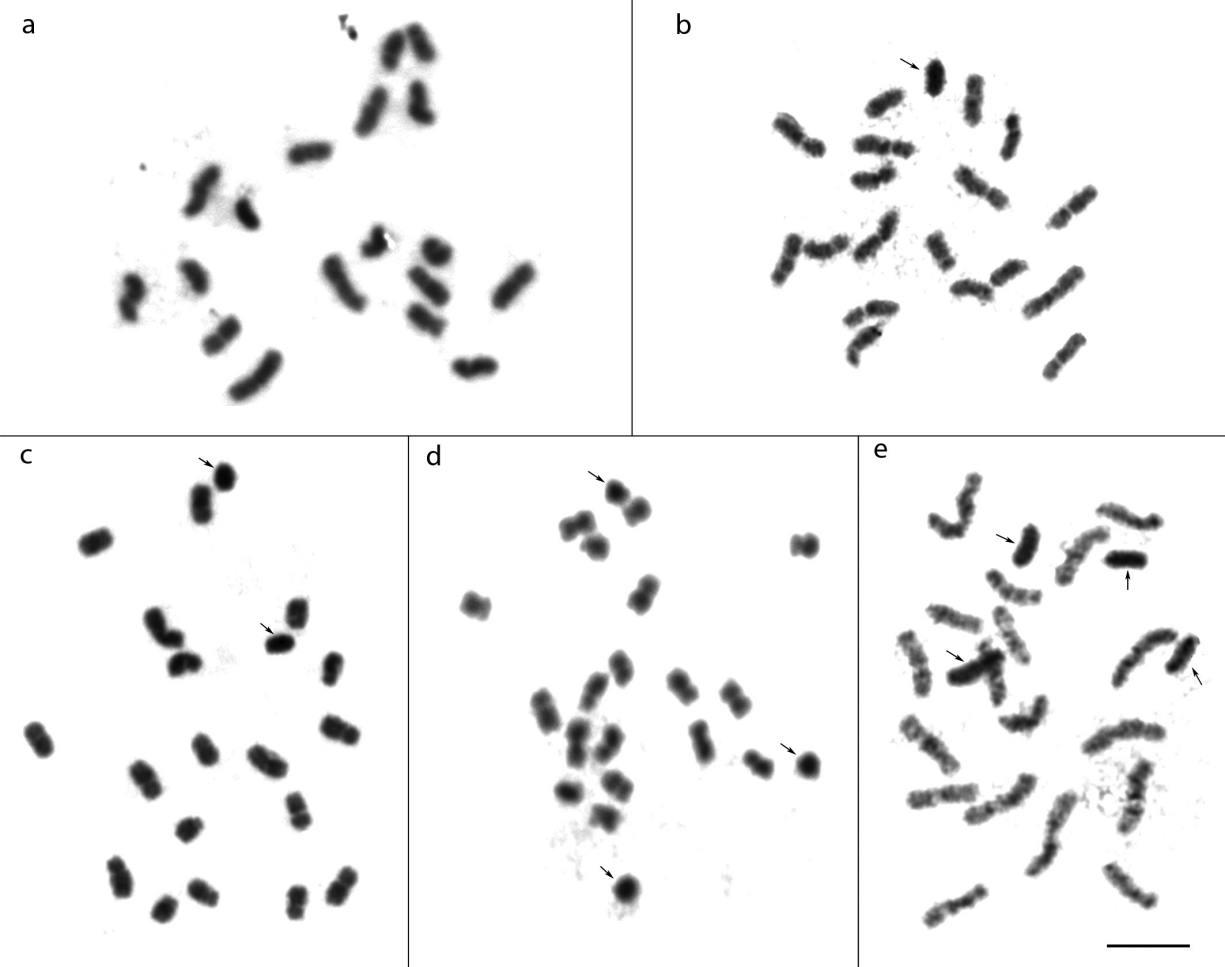
*Meliponaquinquefasciata* metaphases, stained with Giemsa, showing the presence of 0, 1, 2, 3 and 4 B chromosomes (arrows). Scale bar: 5μm.

The chromosome number of *M.quinquefasciata* ranged from 2n = 18 to 22 in females and from n = 9 to 13 in males, as already described by Rocha et al. (2007). Its karyotypic formula was 2K = 10M + 6SM + 2A (Fig. 1). Thus, the typical chromosome number of *M.quinquefasciata* was the same found in most *Melipona* species (2n = 18; Tavares et al. 2017), and numeric variations are due to the presence of 0–4 B chromosomes in females and males (Fig. 2).

In the analysed colonies, the majority of individuals had B chromosomes (Suppl. material 1: Table S1). In samples from Brasília and Luziânia, for example, all females analysed showed at least one B chromosome and only four of the eight analysed males from Luziânia had cells without B chromosomes. Even in these four males, the number of cells with B chromosomes was much higher than the number of cells without them. Similarly, in the colonies from Bicas and Januária, the number of female cells without B chromosomes was very low.

Variations were also observed in the number of B chromosomes between cells of the same individual, between individuals of the same colony, and between colonies from different localities (Fig. 2; Suppl. material 1: Table S1). In samples from Januária, for example, all individuals with B chromosomes had two chromosomes of that kind, while in samples from Brasília, Luziânia and Bicas, individuals with 0, 1, 2, 3 or 4 B chromosomes were found. Intra- and intercolonial variations relating to the presence of B chromosomes have also been described in *P.helleri*, another stingless bee species. In this species, the number of B chromosomes can range from 0–7 between and within colonies and the size of the B chromosome can also vary among colonies from different geographic locations (Costa et al. 1992, Brito et al. 1997, 2005, Tosta et al. 2004, Martins et al. 2014). Likewise, in *M.rufiventris* a small B chromosome was found in a few individuals (males and females) from one of the six colonies analysed (Lopes et al. 2008). Marthe et al. (2010) also described the presence of one B chromosome in some individuals of two colonies of *P.cupira* and Barth et al. (2011) observed that colonies of *Tetragoniscafiebrigi* can harbour individuals with 0, 1 or 2 B chromosomes. Together, our data and these published reports demonstrated that intra- and intercolony variation in the number of B chromosomes is common in stingless bees.

In different individuals and in the analysed colonies as a whole, the number of cells carrying two (411 cells) or three (268 cells) B chromosomes was considerably higher than those that had four B chromosomes (34 cells; Suppl. material 1: Table S1), as previously observed for *P.helleri* (Costa et al. 1992, Brito et al. 1997, Tosta et al. 2004). A more extensive cytogenetic analysis further demonstrated the presence of up to 7 B chromosomes in some *P.helleri* individuals (Martins et al. 2014) and, it is possible that analysis of colonies from other localities may change our perspective on B chromosome numbers for *M.quinquefasciata*. Such analysis could provide insight as to whether there is a mechanism restricting the number of B chromosomes in stingless bees, as originally proposed by Martins et al. (2013). Interestingly, no study has reported a positive or negative effect on fitness related to the presence of different numbers of B chromosomes in this or other *Meliponini* species, as has been found for some other taxa (Camacho 2005).

**Figure 3.**
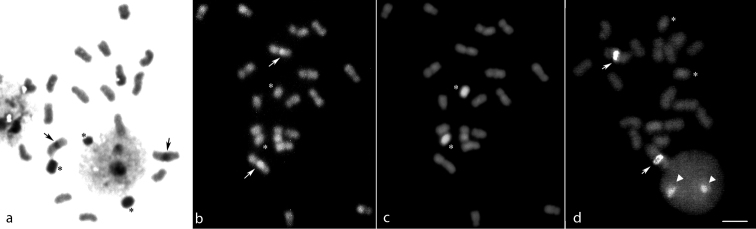
*Meliponaquinquefasciata* metaphase with 2n = 18 + 2Bs submitted to C-banding (**a**), CMA_3_ (**b**) and DAPI (**c**) staining, and to the FISH technique (**d**). The arrows indicate the rDNA location, while asterisks indicate the B chromosomes and arrowheads indicate an interphase nucleus with two signals. Scale bar: 5 μm.

Our data also revealed that, in *M.quinquefasciata*, the heterochromatin, identified by the C-banding technique, was located only in the pericentromeric region of pair 1 (Fig. 3a). Similar results have already been described for other *Melipona* species, such as *M.marginata* Lepeletier, 1836 (Maffei et al. 2001), *M.asilvai* Moure, 1971 (Rocha et al. 2002), *M.compressipes* (Fabricius, 1804) (Rocha et al. 2002), *M.rufiventris*, and *M.mondury* Smith, 1863 (Lopes et al. 2008). Therefore, it was possible to infer that the chromosomes of the A complement of *M.quinquefasciata* had low heterochromatin content. As the genus *Melipona* can be separated in two groups, one with low (Group I) and the other with high (Group II) heterochromatin amounts, *M.quinquefasciata* could be grouped into Group I together with *M.marginata*, *M.quadrifasciata* Lepeletier, 1836, *M.bicolor* Lepeletier, 1836, *M.asilvai*, *M.subnitida* Ducke, 1910, *M.mandacaia* Smith, 1863 and *M.puncticolis* Friese, 1902 (Rocha and Pompolo 1998, Rocha et al. 2002).

However, *M.quinquefasciata* belongs to the subgenus Melikerria Moure, 1992 and species clustered in Group I belong to the subgenera *Melipona* Illiger, 1806 or *Eomelipona* Moure, 1992; Group II clusters species of the subgenera *Melikerria* and *Michmelia* Moure, 1975 (Lopes et al. 2011). Additionally, *M.fasciculata* Smith, 1854 and *M.interrupta* Latreille, 1811, the only other species of the subgenus Melikerria that had their heterochromatin distribution pattern analysed, presented high heterochromatin quantities and were included in Group II (Lopes et al. 2011). This reinforces the need of additional cytogenetic studies concerning species of this subgenus.

By comparison, the B chromosomes of *M.quinquefasciata* were completely heterochromatic, as shown by the C-banding technique (Fig. 3a) and Giemsa staining (Fig. 1), regardless their number in the examined metaphases (Fig. 2). The staining with DAPI confirmed the heterochromatic nature of these chromosomes (Fig. 3c), indicating that, unlike the chromosomes of the A complement, B chromosomes of *M.quinquefasciata* were rich in AT base pairs. Unfortunately, due to their heterochromatic nature, it was not possible to study the morphology of B chromosomes of *M.quinquefasciata* in detail.

CMA_3_ staining and FISH analysis using a 45S rDNA probe confirmed that ribosomal genes were located only in the pericentromeric region of pair 1 in the karyotype of *M.quinquefasciata* (Fig. 3b, d), as already reported for the two colonies analysed by Rocha et al. (2007). The presence of a unique autosome pair with a nucleolus organizer in *M.quinquefasciata* corroborated previous reports about the location of the rDNA clusters in other *Melipona* species, independent of the technique used (Ag-NOR impregnation, CMA_3_ staining or FISH; Rocha et al. 2002, Brito et al. 2003, Lopes et al. 2011, Cunha et al. 2018, Piccoli et al. 2018). This seemed to be the most frequent pattern found in other Meliponini genera (Brito-Ribon et al. 1999, Rocha et al. 2003, Krinski et al. 2010), although the presence of multiple rDNA clusters has also been described (Rocha et al. 2003, Brito et al. 2005, Duarte et al. 2009, Martins et al. 2009, Godoy et al. 2013).

## Conclusion

The results of this study demonstrated that *M.quinquefasciata* has an A complement with a chromosome number characteristic of the *Melipona* genus (2n = 18; n = 9) and a karyotypic formula of 2K = 10M + 6SM + 2A. The numerical variation frequently described for this species might be explained by the presence of a variable number of B chromosomes in individual karyotypes. These chromosomes were found in individuals from different localities and were completely heterochromatic. By comparison, in the chromosomes of the A complement heterochromatin was located only in the pericentromeric region of pair 1, which corresponded to the nucleolus organising region, as demonstrated by CMA_3_ staining and *in situ* hybridisation using a 45S rDNA probe.

## Supplementary materials

Supplementary material 1 Table S1 Data type: species data Explanation note: Sampled localities, number and sex of the individuals of *Meliponaquinquefasciata* analyzed (N) and their cytogenetic characteristics. This is an open access article distributed under the terms of the Creative Commons Attribution License (CC BY 4.0), which permits unrestricted use, distribution, and reproduction in any medium, provided the original author and source are credited.Alexandra Avelar Silva, Marla Piumbini Rocha, Silvia das Graças Pompolo, Lucio Antonio de Oliveira Campos, Mara Garcia Tavares
