# Supplementary material for: Karyotypic description of the stingless bee Meliponaquinquefasciata Lepeletier, 1836 (Hymenoptera, Meliponini) with emphasis on the presence of B chromosomes
Source: Comp Cytogenet. 2018 Nov 9;12(4):471–82. doi: 10.3897/CompCytogen.v12i4.29165 (PMC6240122; doi:10.3897/CompCytogen.v12i4.29165)
Supplement: Supplementary material 1 — Table S1 [file comparative_cytogenetics-12-471-s001.doc]

Table S1 – Sampled localities, number and sex of the individuals of *Melipona* *quinquefasciata* analysed (N) and their cytogenetic characteristics

| Locality | **N** | **No. metaphases analyzed** | **No. metaphases with B chromosomes** | | | | | **Chromosomal numbers found/individual** |
| --- | --- | --- | --- | --- | --- | --- | --- | --- |
|  |  |  | **0B** | **1B** | **2B** | **3B** | **4B** |  |
| Brasília/DF | | | | | | | | |
|  | Female 1 | 7 | - | - | 1 | 6 | - | 2n=20, 21 |
|  | Female 2 | 3 | - | 1 | - | 1 | 1 | 2n=19, 21, 22 |
|  | Female 3 | 1 | - | - | - | 1 | - | 2n=21 |
|  | Female 4 | 1 | - | - | - | 1 | - | 2n=21 |
|  | Female 5 | 1 | - | - | 1 | - | - | 2n=20 |
|  | Female 6 | 3 | - | - | 1 | - | 2 | 2n=20, 22 |
|  | Female 7 | 1 | - | - | - | 1 | - | 2n=21 |
|  | Female 8 | 2 | - | - | - | 2 | - | 2n=21 |
|  | Female 9 | 1 | - | 1 | - | - | - | 2n=19 |
|  | Female 10 | 7 | - | - | 1 | 2 | 4 | 2n=20, 21, 22 |
| Luziânia/GO | | | | | | | | |
|  | Female 1 | 1 | - | - | - | 1 | - | 2n=21 |
|  | Female 2 | 1 | - | - | 1 | - | - | 2n=20 |
|  | Female 3 | 6 | - | - | 1 | 4 | 1 | 2n=20, 21, 22 |
|  | Female 4 | 1 | - | - | 1 | - | - | 2n= 20 |
|  | Female 5 | 2 | - | - | 1 | 1 | - | 2n= 20, 21 |
|  | Female 6 | 10 | - | - | 1 | 9 | - | 2n=20, 21 |
|  | Female 7 | 8 | - | 4 | 4 | - | - | 2n=19, 20 |
|  | Female 8 | 1 | - | 1 | 0 | - | - | 2n=19 |
|  | Female 9 | 17 | - | 2 | 15 | - | - | 2n=19, 20 |
|  | Female 10 | 2 | - | - | 2 | - | - | 2n=20 |
|  | Female 11 | 4 | - | 2 | 2 | - | - | 2n=19, 20 |
|  | Female 12 | 2 | - | - | - | 1 | 1 | 2n=21, 22 |
|  | Female 13 | 1 | - | - | 1 | 0 | 0 | 2n=20 |
|  | Female 14 | 5 | - | 4 | 0 | 0 | 1 | 2n=19 |
|  | Female 15 | 5 | - | - | 2 | 2 | 1 | 2n=20, 21, 22 |
|  | Female 16 | 1 | - | - | 1 | - | - | 2n=20 |
|  | Female 17 | 3 | - | 1 | - | 2 | - | 2n=19, 21 |
|  | Female 18 | 15 | - | 4 | 11 | - | - | 2n=19, 20 |
|  |  |  |  |  |  |  |  |  |
|  | Male 1 | 2 | - | - | - | 1 | 1 | n=12, 13 |
|  | Male 2 | 24 | 3 | 13 | 6 | 2 | - | n=9, 10, 11, 12 |
|  | Male 3 | 24 | 2 | 6 | 15 | 1 | 0 | n=9, 10, 11, 12 |
|  | Male 4 | 2 | - | 1 | 1 | - | - | n=10, 11 |
|  | Male 5 | 12 | 2 | 9 | 1 | - | - | n=9, 10, 11 |
|  | Male 6 | 8 | - | 7 | 1 | - | - | n=10, 11 |
|  | Male 7 | 12 | 3 | 4 | 5 | - | - | n=9, 10, 11 |
|  | Male 8 | 1 | - | 1 | - | - | - | n=10 |
| 1. **Bicas/MG 1** | | | | | | | | |
|  | Female 1 | 12 | - | - | - | 12 | - | 2n=21 |
|  | Female 2 | 10 | - | - | - | 10 | - | 2n=21 |
|  | Female 3 | 10 | - | - | - | - | 10 | 2n=22 |
|  | Female 4 | 10 | - | - | - | 10 | - | 2n=21 |
|  | Female 5 | 10 | - | - | - | 10 | - | 2n=21 |
|  | Female 6 | 10 | - | - | 8 | 2 | - | 2n=20, 21 |
|  | Female 7 | 10 | - | - | - | 10 | - | 2n=21 |
|  | Female 8 | 15 | - | - | 5 | 10 | - | 2n=20, 21 |
|  | Female 9 | 10 | - | - | - | 10 | - | 2n=21 |
|  | Female 10 | 15 | - | - | 4 | 11 | - | 2n=20, 21 |
| 1. **Bicas/MG 2** | | | | | | | | |
|  | Female 1 | 10 | - | - | - | - | 10 | 2n=22 |
|  | Female 2 | 10 | - | - | - | 10 | - | 2n=21 |
|  | Female 3 | 12 | 3 | - | - | 9 | - | 2n=18, 21 |
|  | Female 4 | 10 | - | - | - | 10 | - | 2n=21 |
|  | Female 5 | 18 | - | 3 | 4 | 11 | - | 2n=19, 20, 21 |
|  | Female 6 | 15 | - | - | 11 | 4 | - | 2n=20, 21 |
|  | Female 7 | 10 | - | - | - | 10 | - | 2n=21 |
|  | Female 8 | 14 | - | - | 10 | 4 | - | 2n=20, 21 |
|  | Female 9 | 10 | - | - | 10 | - | - | 2n=20 |
|  | Female 10 | 10 | - | - | 10 | - | - | 2n=20 |
|  |  |  |  |  |  |  |  |  |
| 1. **Bicas/MG 3** | | | | | | | | |
|  | Female 1 | 10 | - | - | - | 10 | - | 2n=21 |
|  | Female 2 | 10 | - | - | - | 10 | - | 2n=21 |
|  | Female 3 | 10 | - | - | - | 10 | - | 2n=21 |
|  | Female 4 | 15 | - | - | - | 13 | 2 | 2n=21, 22 |
|  | Female 5 | 10 | - | - | - | 10 | - | 2n=21 |
|  | Female 6 | 14 | - | - | - | 14 | - | 2n=21 |
|  | Female 7 | 10 | - | - | - | 10 | - | 2n=21 |
|  | Female 8 | 10 | - | - | - | 10 | - | 2n=21 |
|  |  |  |  |  |  |  |  |  |
| **Januária/MG 1** | | | | | | | | |
|  | Female 1 | 14 | - | - | 14 | - | - | 2n=20 |
|  | Female 2 | 12 | - | - | 12 | - | - | 2n=20 |
|  | Female 3 | 10 | - | - | 10 | - | - | 2n=20 |
|  | Female 4 | 10 | - | - | 10 | - | - | 2n=20 |
|  | Female 5 | 12 | - | - | 12 | - | - | 2n=20 |
|  | Female 6 | 10 | - | - | 10 | - | - | 2n=20 |
|  | Female 7 | 10 | - | - | 10 | - | - | 2n=20 |
|  | Female 8 | 13 | - | - | 13 | - | - | 2n=20 |
|  | Female 9 | 10 | - | - | 10 | - | - | 2n=20 |
|  | Female 10 | 10 | - | - | 10 | - | - | 2n=20 |
|  | Female 11 | 12 | - | - | 12 | - | - | 2n=20 |
|  | Female 12 | 10 | - | - | 10 | - | - | 2n=20 |
|  | Female 13 | 13 | 6 | - | 7 | - | - | 2n=18, 20 |
|  | Female 14 | 27 | - | - | 27 | - | - | 2n=20 |
|  |  |  |  |  |  |  |  |  |
| **Januária/MG 2** | | | | | | | | |
|  | Female 1 | 10 | - | - | 10 | - | - | 2n=20 |
|  | Female 2 | 12 | - | - | 12 | - | - | 2n=20 |
|  | Female 3 | 25 | 9 | - | 16 | - | - | 2n=18, 20 |
|  | Female 4 | 11 | 3 | - | 8 | - | - | 2n=18, 20 |
|  | Female 5 | 10 | - | - | 10 | - | - | 2n=20 |
|  | Female 6 | 10 | - | - | 10 | - | - | 2n=20 |
|  | Female 7 | 10 | - | - | 10 | - | - | 2n=20 |
|  | Female 8 | 14 | 4 | - | 10 | - | - | 2n=18, 20 |
|  | Female 9 | 10 | - | - | 10 | - | - | 2n=20 |
|  | Female 10 | 10 | - | - | 10 | - | - | 2n=20 |
| **Total** |  |  | **35** | **64** | **411** | **268** | **34** |  |
